# Supplementary material for: Exosomal microRNA Panels for Detecting Early-Stage Non-Small Cell Lung Cancer
Source: Diagnostics (Basel). 2025 Oct 28;15(21):2735. doi: 10.3390/diagnostics15212735 (PMC12608640; doi:10.3390/diagnostics15212735)
Supplement: Supplementary file 1 [file diagnostics-15-02735-s001.zip › diagnostics-3891966-supplementary.pdf]

Supplementary Figure S1. Distribution of the number and percentage of miRNAs with zero read counts among samples.

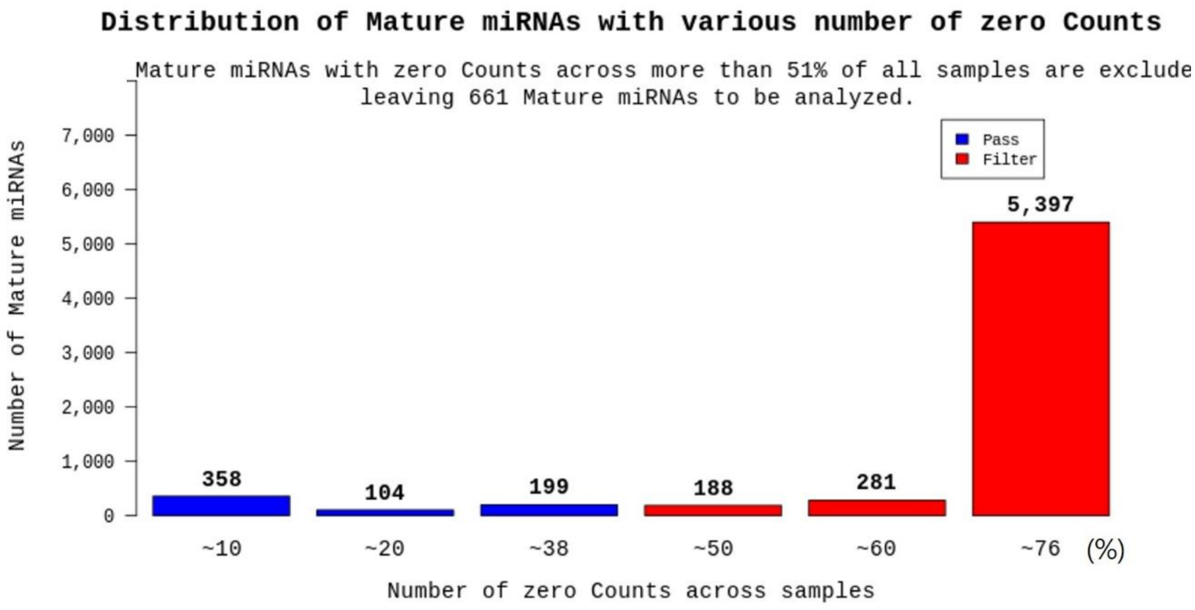

Supplementary Figure S2. Volcano plot from comparative analysis of stages I & II vs benign in the NGS discovery phase. The red dots are miRNAs with FC (I$\log_2$(fold change)I > 2.0 and p < 0.05).

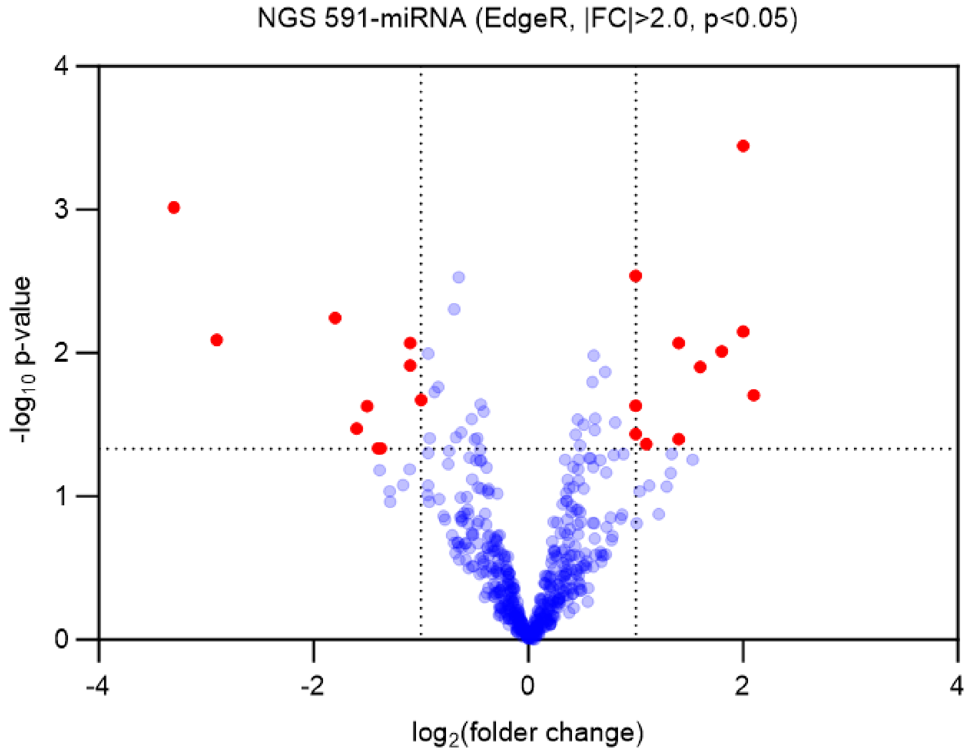

Supplementary Table S1. Serum samples used for RNA quality control and library preparation for NGS analysis.

Sample no. in QC*	Final sample no.	stages	sample name	state	RNA amount			preliminary QC				success of library preparation
					Conc. (ng/ul)	Volume (ul)	Total amount (ug)	bad	maybe OK	good	fairly good	
1	1	I	4314799	RNA	0.549	15	0.008			1		Pass
2	2	I	4110230	RNA	0.617	15	0.009			1		Pass
3	3	I	4090738	RNA	1.553	15	0.023			1		Pass
4	4	I	4654109	RNA	1.095	15	0.016	1				-
5	5	I	4149930	RNA	1.096	15	0.016				1	Pass
6	6	I	4586223	RNA	2.728	15	0.041				1	Pass
7	7	I	4767744	RNA	1.122	15	0.017				1	Pass
8	8	I	4381670	RNA	13.597	15	0.204				1	Pass
9	9	I	4372700	RNA	0.632	15	0.009				1	Pass
10	10	I	4504906	RNA	1.766	15	0.026				1	Pass
11	11	I	4249410	RNA	1.493	15	0.022		1			Pass
12	12	I	4454761	RNA	1.442	15	0.022	1				-
13	13	I	4700074	RNA	1.802	15	0.027			1		Pass
14	14	I	4670761	RNA	0.661	15	0.01				1	Pass
15	15	I	4054929	RNA	1.24	15	0.019			1		Pass
21	16	I	4712962	RNA	0.907	15	0.014				1	Pass
53	17	I	4133089	RNA	1.573	15	0.024				1	Pass
10	18	I	4220719	RNA	0.6	15	0.009	1				-
47	19	I	4716039	RNA	1.013	15	0.015				1	Pass
34	20	I	4750473	RNA	0.767	15	0.012	1				-
26	21	I	4077914	RNA	0.636	15	0.01	1				-
8	22	I	4387656	RNA	0.975	15	0.015			1		Pass
6	23	I	4075789	RNA	1.202	15	0.018		1			Pass
22	24	I	4668396	RNA	0.929	15	0.014	1				-
17	25	I	4025864	RNA	0.478	15	0.007	1				-
29	26	I	4681094	RNA	1.208	15	0.018			1		Pass
2	27	I	4446850	RNA	0.521	15	0.008	1				-
25	28	I	4323136	RNA	0.731	15	0.011	1				-
16	29	II	4161604	RNA	0.89	15	0.013	1				-
17	30	II	4534027	RNA	0.289	15	0.004		1			Pass
18	31	II	4378816	RNA	1.054	15	0.016	1				-
19	32	II	4628743	RNA	0.903	15	0.014	1				-

20	33	II	4669646	RNA	0.944	15	0.014				1	Pass
21	34	II	4203999	RNA	10.323	15	0.155				1	Pass
22	35	II	4648352	RNA	1.768	15	0.027	1				-
23	36	II	4765447	RNA	0.961	15	0.014	1				-
24	37	II	4223348	RNA	2.072	15	0.031			1		Pass
25	38	II	4046017	RNA	1.197	15	0.018		1			Pass
26	39	II	4457685	RNA	2.863	15	0.043	1				-
31	40	II	4532445	RNA	1.681	15	0.025				1	Pass
16	41	II	4791242	RNA	1.235	15	0.019				1	Pass
33	42	II	4178432	RNA	0.59	15	0.009	1				-
3	43	II	4102216	RNA	0.91	15	0.014	1				-
49	44	III	4567274	RNA	0.847	15	0.013				1	Pass
27	45	III	4359432	RNA	1.323	15	0.02	1				-
28	46	III	4588342	RNA	0.945	15	0.014	1				-
29	47	III	4003532	RNA	0.262	15	0.004	1				-
30	48	III	4409174	RNA	3.751	15	0.056				1	Pass
31	49	III	4779112	RNA	7.855	15	0.118				1	Pass
32	50	III	4155585	RNA	0.571	15	0.009				1	Pass
33	51	III	4679389	RNA	1.583	15	0.024			1		Pass
34	52	III	4180209	RNA	1.203	15	0.024			1		Pass
35	53	III	4603564	RNA	1.246	15	0.019			1		Pass
36	54	III	4782336	RNA	1.041	15	0.016			1		Pass
37	55	III	4151690	RNA	0.426	15	0.006	1				-
38	56	III	4642818	RNA	0.325	15	0.005	1				-
39	57	III	4709846	RNA	0.157	15	0.002	1				-
40	58	III	4727772	RNA	0.219	15	0.003	1				-
41	59	III	4323631	RNA	0.276	15	0.004	1				-
27	60	III	4567035	RNA	1.19	15	0.018				1	Pass
50	61	III	4604868	RNA	0.871	15	0.013	1				-
52	62	III	4000392	RNA	0.662	15	0.01	1				-
42	63	III	4097262	RNA	0.718	15	0.011	1				-
13	64	III	4695689	RNA	1.649	15	0.025				1	Pass
46	65	III	4376462	RNA	0.744	15	0.011	1				-
43	66	III	4168620	RNA	0.543	15	0.008	1				-
37	67	III	4068379	RNA	1.441	15	0.022				1	Pass
24	68	III	4268049	RNA	0.668	15	0.01				1	Pass
7	69	III	4728239	RNA	0.363	15	0.005	1				-

51	70	III	4122249	RNA	0.597	15	0.009	1				-
36	71	III	4665304	RNA	1.563	15	0.023			1		Pass
12	72	III	4170658	RNA	0.747	15	0.011	1				-
42	73	IV	4422704	RNA	1.668	15	0.025				1	Pass
43	74	IV	4392052	RNA	1.612	15	0.024				1	Pass
44	75	IV	4241478	RNA	1.008	15	0.015				1	Pass
45	76	IV	4129269	RNA	1.201	15	0.018				1	Pass
46	77	IV	4307478	RNA	0.369	15	0.006	1				-
47	78	IV	4395886	RNA	1.407	15	0.021				1	Pass
48	79	IV	4383927	RNA	2.17	15	0.033			1		Pass
49	80	IV	4301201	RNA	1.724	15	0.026				1	Pass
50	81	IV	4024928	RNA	2.533	15	0.038				1	Pass
51	82	IV	4099268	RNA	1.95	15	0.029			1		Pass
52	83	IV	4476920	RNA	2.273	15	0.034				1	Pass
53	84	IV	4059680	RNA	2.069	15	0.031				1	Pass
54	85	IV	4101703	RNA	1.629	15	0.024			1		Pass
55	86	IV	4499598	RNA	1.346	15	0.02			1		Pass
56	87	IV	4794201	RNA	0.641	15	0.01		1			Pass
32	88	IV	4487788	RNA	1.145	15	0.017			1		**Fail**
14	89	IV	4206735	RNA	0.56	15	0.008	1				-
39	90	IV	4060899	RNA	0.929	15	0.014			1		Pass
35	91	IV	4563283	RNA	1.653	15	0.025				1	Pass
19	92	IV	4445722	RNA	1.375	15	0.021				1	**Fail**
18	93	IV	4369328	RNA	0.966	15	0.014			1		Pass
40	94	IV	4590443	RNA	1.825	15	0.027				1	Pass
38	95	IV	4532993	RNA	1.594	15	0.024				1	Pass
20	96	IV	4572566	RNA	0.885	15	0.013			1		Pass
48	97	IV	4741122	RNA	1.394	15	0.021				1	Pass
57	98	benign	4680558	RNA	0.997	15	0.015				1	Pass
58	99	benign	4640924	RNA	1.914	15	0.029				1	Pass
59	100	benign	4771457	RNA	0.676	15	0.01	1				-
60	101	benign	4103192	RNA	0.916	15	0.014			1		Pass
61	102	benign	4362994	RNA	0.301	15	0.005	1				-
62	103	benign	4334375	RNA	1.686	15	0.025			1		Pass
63	104	benign	4172239	RNA	2.341	15	0.035				1	Pass
64	105	benign	4701318	RNA	0.468	15	0.007	1				-
65	106	benign	4349620	RNA	0.636	15	0.01	1				-
66	107	benign	4424174	RNA	2.414	15	0.036			1		Pass

67	108	benign	4586291	RNA	1.96	15	0.029				1	Pass
68	109	benign	4408692	RNA	1.729	15	0.026			1		Pass
69	110	benign	4391575	RNA	1.579	15	0.024				1	Pass
70	111	benign	4500471	RNA	1.907	15	0.029				1	**Fail**
71	112	benign	4474392	RNA	1.339	15	0.02			1		Pass
44	113	benign	4314471	RNA	0.728	15	0.011			1		Pass
30	114	benign	4368948	RNA	0.595	15	0.009	1				-
9	115	benign	4644712	RNA	1.267	15	0.019			1		Pass
54	116	benign	4708279	RNA	1.011	15	0.015	1				-
11	117	benign	4332326	RNA	0.575	15	0.009	1				-
23	118	benign	4052985	RNA	0.763	15	0.011	1				-
45	119	benign	4616155	RNA	0.642	15	0.01	1				-
1	120	benign	4236094	RNA	1.374	15	0.021			1		Pass
4	121	benign	4271568	RNA	0.724	15	0.011			1		Pass
28	122	benign	4389393	RNA	0.53	15	0.008	1				-
41	123	benign	4282347	RNA	0.322	15	0.005	1				-
5	124	benign	4006619	RNA	1.294	15	0.019				1	Pass
15	125	benign	4673798	RNA	0.728	15	0.011				1	Pass

*The serum samples for NGS analysis were provided in two separate supplies. To differentiate those samples, two different colors were used, the blue for the first supply and the red for the 2nd supply.

Supplementary Table S2. Expression profiles of the miRNAs with significantly differentiated expressions (FC>2.0 & p<0.05) of the comparative analysis in each respective stage vs benign in the discovery phase. (FC values of the down-regulated miRNAs are in red colors.)

Stage-I vs Benign	FC>2.0	p<0.05
hsa-miR-128-1-5p	3.0	0.0267
hsa-miR-140-5p	2.1	0.0269
hsa-miR-150-5p	2.1	0.0044
hsa-miR-190a-5p	2.1	0.0220
hsa-miR-193b-3p	2.6	0.0359
hsa-miR-1972	2.5	0.0125
hsa-miR-1976	3.0	0.0020
hsa-miR-199b-5p	2.1	0.0201
hsa-miR-19a-3p	2.1	0.0047
hsa-miR-202-3p	3.9	0.0069
hsa-miR-2355-5p	2.2	0.0240
hsa-miR-301a-3p	2.1	0.0163
hsa-miR-301b-3p	4.4	0.0000
hsa-miR-32-5p	2.4	0.0092
hsa-miR-337-3p	2.9	0.0390
hsa-miR-3605-3p	2.6	0.0178
hsa-miR-4444	5.6	0.0034
hsa-miR-4732-3p	2.1	0.0189
hsa-miR-4787-3p	2.5	0.0309
hsa-miR-551a	2.9	0.0338
hsa-miR-5585-3p	3.4	0.0075
hsa-miR-610	2.6	0.0381
hsa-miR-642b-3p	2.1	0.0202
hsa-miR-6807-5p	3.2	0.0045
hsa-miR-6873-3p	2.2	0.0425
hsa-miR-769-3p	3.3	0.0048
hsa-miR-7854-3p	2.1	0.0221

Stage-II vs Benign	FC>2.0	p<0.05
hsa-miR-181c-5p	2.5	0.0196
hsa-miR-194-5p	2.3	0.0149
hsa-miR-1972	2.2	0.0285
hsa-miR-1976	3.1	0.0038
hsa-miR-202-3p	5.6	0.0109
hsa-miR-301b-3p	3.1	0.0323
hsa-miR-3150b-3p	3.9	0.0187
hsa-miR-3605-3p	2.6	0.0288
hsa-miR-4449	2.1	0.0210
hsa-miR-652-5p	2.7	0.0251
hsa-miR-885-5p	4.4	0.0026

Stage-III vs Benign	FC>2.0	p<0.05
hsa-miR-122-3p	11.6	0.0001
hsa-miR-1260a	2.2	0.0058
hsa-miR-1268a	2.2	0.0116
hsa-miR-1270	2.6	0.0404
hsa-miR-1290	2.6	0.0038
hsa-miR-132-3p	2.5	0.0270
hsa-miR-155-5p	2.6	0.0302
hsa-miR-194-5p	2.0	0.0223
hsa-miR-1976	2.2	0.0235
hsa-miR-19a-3p	2.1	0.0185
hsa-miR-202-3p	3.3	0.0193
hsa-miR-223-3p	2.2	0.0084
hsa-miR-2278	3.0	0.0021
hsa-miR-27a-5p	2.0	0.0410
hsa-miR-29a-3p	2.2	0.0114
hsa-miR-301a-3p	2.7	0.0064
hsa-miR-301b-3p	3.5	0.0019
hsa-miR-32-5p	2.2	0.0107
hsa-miR-369-3p	2.6	0.0274
hsa-miR-4429	2.7	0.0067
hsa-miR-4449	2.3	0.0039
hsa-miR-455-3p	7.6	0.0013
hsa-miR-4749-5p	4.3	0.0137
hsa-miR-4750-5p	2.6	0.0339
hsa-miR-4772-5p	3.6	0.0170
hsa-miR-491-5p	2.1	0.0202
hsa-miR-551a	2.8	0.0317
hsa-miR-5585-3p	3.1	0.0058
hsa-miR-627-5p	2.7	0.0037
hsa-miR-660-5p	2.1	0.0088
hsa-miR-6855-5p	4.9	0.0025
hsa-miR-940	2.7	0.0044

Stage-IV vs Benign	FC>2.0	p<0.05
hsa-miR-1246	6.1	0.0000
hsa-miR-1273c	2.2	0.0027
hsa-miR-1285-3p	2.2	0.0023
hsa-miR-1290	4.5	0.0000
hsa-miR-181c-5p	2.6	0.0016
hsa-miR-193b-3p	2.3	0.0226
hsa-miR-196b-5p	2.2	0.0076
hsa-miR-1972	2.2	0.0095
hsa-miR-200b-5p	3.6	0.0178
hsa-miR-205-5p	4.8	0.0011
hsa-miR-27b-5p	2.1	0.0430
hsa-miR-301b-3p	2.6	0.0137
hsa-miR-3158-5p	2.7	0.0030
hsa-miR-375-3p	6.7	0.0014
hsa-miR-378h	2.8	0.0088
hsa-miR-4646-5p	2.0	0.0146
hsa-miR-4732-5p	2.0	0.0017
hsa-miR-4753-5p	2.7	0.0312
hsa-miR-4787-3p	6.1	0.0018
hsa-miR-483-5p	3.4	0.0037
hsa-miR-5100	2.8	0.0280
hsa-miR-518e-5p	2.8	0.0487
hsa-miR-519a-5p	2.8	0.0486
hsa-miR-519b-5p	2.8	0.0485
hsa-miR-519c-5p	2.8	0.0484
hsa-miR-522-5p	2.8	0.0483
hsa-miR-523-5p	2.8	0.0483
hsa-miR-543	2.7	0.0216
hsa-miR-5585-3p	3.0	0.0135
hsa-miR-610	2.7	0.0484
hsa-miR-6125	2.9	0.0222
hsa-miR-627-5p	2.3	0.0164
hsa-miR-6804-5p	2.5	0.0199
hsa-miR-6837-5p	3.5	0.0240
hsa-miR-6873-3p	2.7	0.0137
hsa-miR-762	2.6	0.0325
hsa-miR-765	4.3	0.0036

Supplementary Table S3. miRNAs identified based on the Mann–Whitney U test (p<0.05).

no	**miRNAs (stages I & II vs. benign)**	**p_{M-W} <0.05**	*no*	**miRNAs (stages I & II vs. benign)**	**p_{M-W} <0.05**
1	hsa-miR-130b-3p	0.001	*33*	hsa-miR-532-3p	0.019
2	hsa-miR-1976	0.001	*34*	hsa-miR-769-3p	0.019
3	hsa-miR-301b-3p	0.001	*35*	hsa-miR-3615	0.02
4	hsa-miR-130a-3p	0.002	*36*	hsa-miR-128-1-5p	0.021
5	hsa-miR-369-3p	0.002	*37*	hsa-miR-196b-5p	0.021
6	hsa-miR-24-3p	0.003	*38*	hsa-miR-19a-3p	0.022
7	hsa-miR-199a-3p	0.004	*39*	hsa-miR-484	0.022
8	hsa-miR-199b-3p	0.004	*40*	hsa-miR-151a-3p	0.023
9	hsa-miR-32-5p	0.004	*41*	hsa-miR-32-3p	0.023
10	hsa-miR-497-5p	0.004	*42*	hsa-miR-223-5p	0.025
11	hsa-miR-126-3p	0.005	*43*	hsa-miR-6807-5p	0.026
12	hsa-miR-1285-5p	0.005	*44*	hsa-miR-610	0.027
13	hsa-miR-7-5p	0.005	*45*	hsa-miR-181c-5p	0.028
14	hsa-miR-324-3p	0.006	*46*	hsa-miR-144-3p	0.029
15	hsa-miR-423-3p	0.006	*47*	hsa-miR-2355-3p	0.029
16	hsa-miR-1292-5p	0.007	*48*	hsa-miR-26b-5p	0.029
17	hsa-miR-150-5p	0.008	*49*	hsa-miR-106a-5p	0.033
18	hsa-miR-4286	0.008	*50*	hsa-miR-1273c	0.033
19	hsa-miR-342-3p	0.01	*51*	hsa-miR-128-3p	0.033
20	hsa-miR-424-5p	0.011	*52*	hsa-miR-3605-3p	0.033
21	hsa-miR-574-5p	0.012	*53*	hsa-miR-197-3p	0.035
22	hsa-miR-874-5p	0.012	*54*	hsa-miR-125a-5p	0.037
23	hsa-miR-20a-5p	0.015	*55*	hsa-miR-142-5p	0.037
24	hsa-miR-4685-3p	0.015	*56*	hsa-miR-3150b-3p	0.039
25	hsa-miR-762	0.015	*57*	hsa-miR-101-3p	0.04
26	hsa-miR-335-5p	0.016	*58*	hsa-miR-199b-5p	0.04
27	hsa-miR-5585-3p	0.017	*59*	hsa-miR-6873-3p	0.04
28	hsa-let-7d-5p	0.018	*60*	hsa-miR-652-5p	0.041
29	hsa-miR-374a-5p	0.018	*61*	hsa-miR-326	0.042
30	hsa-miR-106b-5p	0.019	*62*	hsa-miR-10527-5p	0.046
31	hsa-miR-202-3p	0.019	*63*	hsa-miR-190a-5p	0.046
32	hsa-miR-21-5p	0.019	*64*	hsa-miR-370-3p	0.047

Supplementary Table S4. Fourteen miRNAs identified based on the analytical criteria of both Edge-R (FC>2.0 and p <0.05) and Mann–Whitney U test (p <0.05). The FC values of the down-regulated miRNAs are in red.

miRNAs	edgeR				Mann-Whitney U-test
	stages I, II vs benign		stages I~IV vs benign		stages I, II vs benign
	FC>2.0	p<0.05	FC	p	p <0.05
hsa-miR-128-1-5p	3.1	0.0198	2.7	0.0244	0.021
hsa-miR-150-5p	2.0	0.0029	1.6	0.0173	0.008
hsa-miR-190a-5p	2.1	0.0123	1.9	0.0105	0.046
hsa-miR-1976	3.0	0.0004	2.4	0.0012	0.001
hsa-miR-202-3p	4.3	0.0010	3.3	0.0005	0.019
hsa-miR-301b-3p	3.9	0.0001	3.3	0.0002	0.001
hsa-miR-3605-3p	2.6	0.0126	2.2	0.0257	0.033
hsa-miR-369-3p	2.6	0.0338	2.1	0.0455	0.002
hsa-miR-5585-3p	3.0	0.0071	3.0	0.0026	0.017
hsa-miR-610	2.5	0.0234	2.2	0.0245	0.027
hsa-miR-6807-5p	2.8	0.0057	1.9	0.0637	0.026
hsa-miR-6873-3p	2.1	0.0431	2.1	0.0394	0.040
hsa-miR-769-3p	2.8	0.0098	2.3	0.0377	0.019
hsa-miR-874-5p	2.0	0.0234	1.6	0.1073	0.012

Supplementary Table S5. AUCs of the different sets of miRNA panels.

(A) AUCs from five-miRNA panels

up miRNAs	miR-150-5p	miR-150-5p	miR-150-5p	miR-150-5p	miR-150-5p
down miRNAs	miR-301b-3p<br>miR-369-3p<br>miR-497-5p<br>miR-610	miR-1976<br>miR-369-3p<br>miR-497-5p<br>miR-610	miR-1976<br>miR-301b-3p<br>miR-497-5p<br>miR-610	miR-1976<br>miR-301b-3p<br>miR-369-3p<br>miR-610	miR-1976<br>miR-301b-3p<br>miR-369-3p<br>miR-497-5p
**AUC**	**0.88**	**0.8171**	**0.7957**	**0.81**	**0.9343**

(B) AUCs from four-miRNA panels

up miRNA	miR-150-5p	miR-150-5p	miR-150-5p	miR-150-5p
down miRNAs	miR-301b-3p<br>miR-369-3p<br>miR-497-5p	miR-1976<br>miR-369-3p<br>miR-497-5p	miR-1976<br>miR-301b-3p<br>miR-497-5p	miR-1976<br>miR-301b-3p<br>miR-369-3p
**AUC**	**0.9329**	**0.92**	**0.8843**	**0.9114**

(C) AUCs from three-miRNA panels

up miRNA	miR-150-5p	miR-150-5p	miR-150-5p
down miRNAs	miR-369-3p<br>miR-497-5p	miR-301b-3p<br>miR-497-5p	miR-301b-3p<br>miR-369-3p
**AUC**	**0.9186**	**0.9414**	**0.8986**

(D) AUCs from two-miRNA panels

up miRNA	miR-150-5p	miR-150-5p
down miRNAs	miR-301b-3p	miR-497-5p
**AUC**	**0.9038**	**0.9113**

Explanation for the four different tables (Tables (A), (B), (C) and (D)).

(A) Based on the six miRNAs, miR-1976, miR-150, miR-1976, miR-301b-3p, miR-369-3p and miR-497-5p, five different sets of panels composed of five miRNAs were arranged and AUCs were obtained to be compared. Among the five different panels, the highest AUC was from the panel composed of miR-150, miR-1976, miR-301b-3p, miR-369-3p and miR-497-5p.

(B) Now, choosing the best AUC from the five-miRNA panel, four different sets of miRNA panels were arranged and their AUCs were calculated. Among the four different panels, the highest AUC (0.9329) resulted from the panel comprised of miR-150, miR-301b-3p, miR-369-3p and miR-497-5p.

(C) Now, using the best AUC from the four-miRNA panel, three different sets of miRNA panels were arranged and their AUCs were calculated. Among the three panels, the highest AUC (0.9414) resulted from the panel comprised of miR-150, miR-301b-3p, and miR-497-5p.

(D) Now, using the best AUC from the three-miRNA panel, two different sets of miRNA panels were arranged and their AUCs were calculated. The AUC values of these two sets of panels were similar with AUC values centered around 0.9.

Rationale for selecting the two best miRNA panels: From the four tables, four respective best sets of miRNA panels can be selected. The best AUC value is from the three-miRNA panel of miR-150, miR-301b-3p and miR-497-5p. Although the second best one is from the five-miRNA panel of miR-150, miR-1976, miR-301b-3p, miR-369-3p and miR-497-5p (AUC 0.9343), the third-ranked panel is from the four-miRNA panel of miR-150, miR-301b-3p, miR-369-3p, and miR-497-5p with only negligibly minute difference of 0.0004. So, the four-miRNA panel was preferred to the five-miRNA panel due to smaller number of constituting miRNAs.

Supplementary Table S6. Target genes interaction characteristics of the intimately networked miRNAs

**Networked miRNA**	**Target genes**																			
	BCL2	BCL6	BIRC5	BMI1	CDC25A	E2F1	E2F3	ERBB2	HDGF	HMGB1	IGF1R	LRP6	PDCD4	PTEN	RUNX2	SMAD7	SP1	TP63	VEGFA	ZEB1
hsa-miR-128-1-5p				●			●													
hsa-miR-150-5p			●														●		●	●
hsa-miR-202-3p												●	●							
hsa-miR-205-5p	●	●				●		●		●				●	●				●	●
hsa-miR-21-5p	●	●		●	●	●		●		●	●	●	●	●		●	●	●	●	
hsa-miR-301b-3p														●				●		
hsa-miR-497-5p	●		●		●		●		●		●				●	●				
hsa-miR-610									●											

Supplementary Table S7. Enrichment analysis of the 15 miRNAs identified in the discovery phase in relation to lung diseases.

lung diseases (15 miR, miEAA 2.0)	p-value	p-adjusted	no	related miRNAs
Lung squamous cell carcinoma	< 0.001	< 0.001	13	hsa-miR-202-3p; hsa-miR-769-3p; hsa-miR-497-5p; hsa-miR-21-5p; hsa-miR-6873-3p; hsa-miR-5585-3p; hsa-miR-150-5p; hsa-miR-301b-3p; hsa-miR-3605-3p; hsa-miR-369-3p; hsa-miR-1976; hsa-miR-190a-5p; hsa-miR-205-5p
Lung adenocarcinoma	< 0.001	< 0.01	12	hsa-miR-202-3p; hsa-miR-610; hsa-miR-769-3p; hsa-miR-497-5p; hsa-miR-21-5p; hsa-miR-5585-3p; hsa-miR-150-5p; hsa-miR-301b-3p; hsa-miR-1976; hsa-miR-128-1-5p; hsa-miR-190a-5p; hsa-miR-205-5p
Lung disease	< 0.01	0.031	7	hsa-miR-202-3p; hsa-miR-610; hsa-miR-769-3p; hsa-miR-497-5p; hsa-miR-21-5p; hsa-miR-301b-3p; hsa-miR-205-5p
Lung small cell carcinoma	< 0.02	0.033	6	hsa-miR-202-3p; hsa-miR-497-5p; hsa-miR-21-5p; hsa-miR-150-5p; hsa-miR-301b-3p; hsa-miR-205-5p
Carcinoma lung non-small-cell	< 0.03	0.033	4	hsa-miR-202-3p; hsa-miR-21-5p; hsa-miR-1976; hsa-miR-205-5p
Non-small cell lung cancer	< 0.04	0.045	3	hsa-miR-21-5p; hsa-miR-150-5p; hsa-miR-205-5p

Supplementary Text. An example of the UDR calculation used to obtain ROC curves for the 6 miRNA-panel in the validation phase.

The validation sample ID 04380766 was used to describe the UDR calculation. The values of ΔCt for miR-150-5p, miR-21-5p, miR-301b-3p, miR-369-3p, miR-497-5p, and miR-610 are 13.17, 13.13, 19.13, 15.24, 17.31, and 30.52, respectively. The fold changes (FCs) of miR-150-5p, miR-21-5p, miR-301b-3p, miR-369-3p, miR-497-5p, and miR-610 in the stages I and II group compared with the benign group were 1.64, 0.46, 0.53, 0.31, 0.39, and 0.30, respectively.

Then, for the stages I and II group compared with the benign group, the value used to obtain an ROC curve for the sample 04380766 (benign) can be calculated as follows:

average ΔCt of the upregulated miRNAs = 13.17

average ΔCt of the downregulated miRNAs = (13.13 + 19.13 + 15.24 + 17.31 + 30.52)/5 = 18.08

UDR value for a sample = (average of ΔCt values for the upregulated miRNAs)/(average of ΔCt values for the downregulated miRNAs) = 13.17/18.08 = 0.73
